# Cleaner fish escape salmon farms and hybridize with local wrasse populations

**DOI:** 10.1098/rsos.171752

**Published:** 2018-03-21

**Authors:** Ellika Faust, Kim Tallaksen Halvorsen, Per Andersen, Halvor Knutsen, Carl André

**Affiliations:** 1 Department of Marine Sciences - Tjärnö, University of Gothenburg, 45296 Strömstad, Sweden; 2 Institute of Marine Research, Austevoll Research Station, Storebø, Norway; 3 Marine senior advisor Nord-Trøndelag, 7770 Flatanger, Norway; 4 Institute of Marine Research, Flødevigen, Norway; 5 Centre for Ecological and Evolutionary Synthesis, Department of Biosciences, University of Oslo, Oslo, Norway; 6 Centre for Coastal Research, University of Agder, Kristiansand, Norway

**Keywords:** aquaculture, wrasse, sea lice, hybrid, RAD, salmon

## Abstract

The genetic impact of farmed fish escaping aquaculture is a highly debated issue. However, non-target species, such as cleaner fish used to remove sea lice from farmed fish, are rarely considered. Here, we report that wild corkwing wrasse (*Symphodus melops*), which are transported long distances to be used as cleaner fish in salmon farms, escape and hybridize with local populations. Recently, increasing numbers of corkwing wrasse have been reported in Flatanger in Norway, north of its described distribution range, an area heavily relying on the import of cleaner fish from Skagerrak. Using genetic markers identified with 2bRAD sequencing, we show that, although the Flatanger population largely is a result of a northward range expansion, there is also evidence of considerable gene flow from southern populations in Skagerrak and Kattegat. Of the 40 corkwing wrasses sampled in Flatanger, we discovered two individuals with clear southern genotypes, one first-generation hybrid, and 12 potential second-generation hybrids. In summary, we provide evidence that corkwing wrasse escape from fish farms and hybridize with local populations at the leading edge of an ongoing range expansion. Although the magnitude and significance of escapees warrant further investigation, these results should be taken into consideration in the use of translocated cleaner fish.

## 1. Introduction

Marine species display a range of levels of genetic divergence among populations, from panmictic species to species with marked genetic structure, as a consequence of reduced gene flow, genetic drift and/or local adaptation [1]. Transferring individuals between spatially separated populations that are genetically distinct is likely to result in genetic changes to native populations. Such changes could involve shifts in allelic composition, loss of genetic variation, erosion of local adaptation and/or breakdown of population structure [2]. Human-mediated releases of genetically different individuals to native populations are increasingly common. Farmed fish escaping aquaculture is a serious threat to wild fish populations, through competition, transfer of diseases and pathogens, and gene flow through interbreeding [3]. There are many examples from open-pen farming of salmonids, where escapees have hybridized with local river populations, leading to genetic swamping and reduced fitness [4,5].

Salmon farming may also promote inadvertent gene flow in populations of species of wrasses (Labridae) in Norway and the UK, where wild wrasses are caught and used as cleaner fish to mitigate the increasing problems of sea lice infestations in the farmed salmon [6,7]. These wrasses are relatively small predatory fish, typically abundant at shallow depths on rocky coastlines in northern Europe. They had little to no commercial value until their function as cleaner fish in captivity was discovered and applied in the late 1980s [8–10]. The use of cleaner fish increased drastically in 2010 as a result of sea lice evolving resistance to the most widely used pharmaceutical treatments [11]. In Norway, the national landings of wrasse have now surpassed 20 million fish annually [12]. However, in mid-Norway, the demand for cleaner fish exceeds the supply from local stocks, and wild-caught wrasses are imported from southern Norway and western Sweden, areas where salmon farming is absent [13]. Similarly, in the UK, most salmon farms are situated in Scotland, but due to local supply not meeting the demand, an estimated 1 million wrasse are harvested in southwestern England annually for live transport to Scotland [14,15]. Furthermore, the UK wrasse fishery is largely undocumented, and the records of landed wrasse are rarely specified by species, only under a generic wrasse code. The lack of data on species composition and landings makes it difficult to assess the impact of the wrasse fishery. This is a concern that has received increasing attention in recent years, resulting in restrictions on wrasse fisheries in southwestern UK by regional Inshore Fisheries and Conservation Authorities (IFCA) [15–17].

In Norway, two species, the goldsinny wrasse (*Ctenolabrus rupestris*) and the corkwing wrasse (*Symphodus melops*), are the most commonly used wild cleaner fish, with 39% and 52% of the total Norwegian official landings 2016, respectively (Norwegian directorate of Fisheries; https://www.fiskeridir.no/Yrkesfiske/Tema/Leppefiske/Registrert-uttak-av-leppefisk-i-2017). A recent study found relatively low genetic divergence between wild goldsinny populations in farming areas in mid-Norway and populations in southern Norway and Sweden, suggesting inadvertent gene flow [18]. In contrast to the goldsinny, which generally shows weak population structure, the corkwing has highly differentiated populations in Scandinavia with a strong genetic break between southern and western Norway and overall lower genetic diversity in the southern area [19]. The difference in population structure between the two species could be related to differences in population connectivity caused by distinct reproductive strategies: the goldsinny is a broadcast spawner with a fraction of the eggs being pelagic, while the corkwing lay benthic eggs in seaweed nests [6,20,21]. Furthermore, southern corkwing populations have been found to grow faster and mature earlier than the populations further north [22], which aligns with the genetic break [19]. Thus, if corkwing with southern origin escapes and hybridizes with local populations further north, we could expect to see changes in genotype composition with possible phenotypic effects.

The corkwing's northern distribution range was earlier reported to extend to the Trondheims Fjord in mid-Norway. In the Flatanger municipality, North Trøndelag county, 130 km further north, no corkwing was found during extensive field surveys of wrasse in the 1990s [23]. However, in recent years, occasional observations of corkwing have been reported in North Trøndelag (but not further north; Norwegian Fishermen's Sales Organization 2016, personal communication), indicating a recent northward range expansion. Knutsen *et al*. [24] proposed that the current increase in abundance in southern Scandinavia is a result of population growth due to rising temperatures, and that the predicted rise in sea temperature could facilitate a northward expansion. The other possibility would be that this northward expansion is a direct result of wrasse escaping from the salmon pens through tears in the net, small fish slipping through the mesh [13,25] or intentional release at the end of the season [26].

Here, we investigate the origin of wild corkwing wrasse captured in Flatanger, amid salmon farms where wrasses are currently used as cleaner fish and rely heavily on the import of wrasse from southern Norway and Sweden. We used the restriction-site-associated DNA (RAD) sequencing method 2b-RAD [27] to simultaneously discover and genotype thousands of SNPs (single nucleotide polymorphisms) across the entire genome [24]. Our objective is to investigate whether the wild corkwing in Flatanger represents: (i) the leading edge of an ongoing northward range expansion [24], (ii) escaped wrasse from aquaculture with origin from Skagerrak and Kattegat or (iii) a mix of both. To answer these questions, we compare SNPs from corkwing wrasse collected in Flatanger with wrasse collected: (i) in western Norway, where wrasse is harvested but used locally, and (ii) further south on the Skagerrak–Kattegat coast, where all wrasses are harvested for live transport to salmon farms in mid- and northern Norway.

## 2. Material and methods

### 2.1. Sampling and DNA extraction

**Figure 1.**
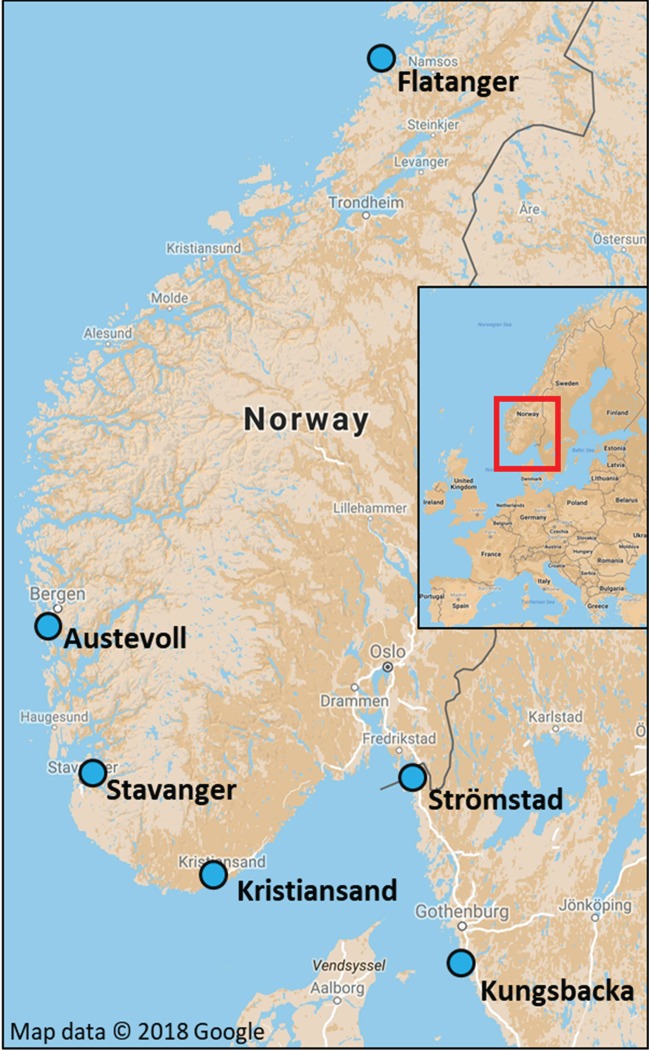
Map of sampling locations. Kristiansand, Strömstad and Kungsbacka are referred to as ‘southern population’, Austevoll and Stavanger as ‘western population’ and Flatanger as ‘mid-Norwegian population’.

With the help of commercial fishermen and local researchers, we collected corkwing wrasse from Flatanger in mid-Norway; from two locations in western Norway: Austevoll and Stavanger (western population); and from three locations at the Skagerrak–Kattegat coast: Kristiansand, Strömstad and Kungsbacka (southern population) (figure 1). Fin clips from forty individuals per location were taken in June–October 2016 and stored in 96% ethanol until further analysis. For fish sampled in Flatanger, we dissected otoliths and aged them by counting annual growth increments following Halvorsen *et al*. [22]. Additional sampling information, such as coordinates and sampling location in relation to salmon farms, can be found in electronic supplementary material, S1 and S2.

Genomic DNA was extracted using DNeasy^®^ Blood & Tissue Kit (Qiagen) with optional RNAse treatment (200 mg RNAse), and purified and concentrated with standard ethanol/isopropanol precipitation. DNA quantity and quality (i.e. presence of contaminants, degradation etc.) were assessed using Qubit^®^ ds DNA BR AssayKit (Invitrogen–ThermoFisher Scientific) and on a 1% agarose gel. 2b-RAD libraries were prepared following a protocol modified from Matz & Aglyamova [28], available in a dedicated GitHub repository (https://github.com/ellikafaust/S.melopsPopGen). All individual DNA samples were tagged with unique barcodes and then pooled in sets of 24 per sequencing lane, including technical replicates of four individuals to control for methodical artefacts. Pooling was done by sampling site, where each sample (40 individuals) was divided in two independent pools that were sequenced in separate lanes. This was done to minimize the risk of mixing up samples during library preparation, while having two independent pools to account for any lane bias. Single-read, 50 bp target length sequencing on Illumina HiSeq2500 platform was conducted at the SNP&SEQ Technology Platform in Uppsala, Uppsala University.

### 2.2. Bioinformatics

The bioinformatic analysis of the DNA sequences followed a modified de novo pipeline from Pierre de Wit [29] using scripts developed by Mikhail Matz (scripts and manual available at https://github.com/z0on/2bRAD_denovo). First, low-quality reads and redundant sequences (i.e. restriction sites and duplicates) were removed. Remaining fragments were then clustered into rad tags, allowing up to three mismatches among reads (identity threshold 91%) and with a minimum depth of 20 reads. Individual genotypes were called, following the criteria of Mindp = 5 (min depth for calling a homozygote), hetero = 0.8 (max fraction of heterozygotes allowed), aobs = 20 (min number of times allele has to be observed across all samples) and strbias = 20 (strand bias cut-off). Four technical replicates per lane were used to control for methodical artefacts using the recalibrateSNPs.pl script. Variants that had been identically genotyped between the replicates were used as reference for non-parametric quality recalibration of all variants, estimating their probability of being ‘true’ SNPs. Loci with recalibrated quality below 20 and alleles with quality below 20 were removed. Only variants with less than 75% heterozygotes and less than 50% missing data were kept for thinning (removing) of the dataset. SNPs occurring on the same RAD-tag were removed, leaving only the SNP with the highest minor allele frequency (MAF) in each RAD-tag. Technical replicates and poorly sequenced individuals (individuals with more than 50% missing data) were removed. Finally, we removed loci that were missing in more than 30% of the individuals or with a global MAF below 1%. Initially, different levels of minor allele frequency (maf 0%, 1% and 5%) were tested. As the different datasets did not change the outcome of the analyses (data not shown), we only present results from loci with maf > 1%, maintaining the most number of loci, while still removing genotyping errors and uninformative polymorphisms [30]. Data conversions between different software technologies were done using PGD spider [31].

### 2.3. Statistical analysis

#### 2.3.1. Population diversity and differentiation

We used the R package diveRsity [32] in R v. 3.3.2 [33] to calculate observed and expected heterozygosity for each locus in the different samples. Whether observed heterozygosity (*H*o) values deviated from expected heterozygosity (*H*e) was assessed by calculating *F*_IS_ according to Weir & Cockerham [34]. Deviations from Hardy–Weinberg (HW) proportions were estimated with exact tests, with *p*-values calculated according to the complete enumeration method [35] and adjusted for multiple testing using false discovery rate (FDR) correction [36]. Loci that deviated (*q* < 0.05) from HW proportions in more than one of the samples were subsequently removed. Weir & Cockerham's *F*_ST_ was estimated for each population pair and over all samples using diveRsity. Statistical significance of *F*_ST_ values was assessed using Fisher's exact probability test with 5000 Monte Carlo replicates, followed by FDR correction.

#### 2.3.2. Individual-based clustering

Missing genotypes can induce patterns of similarity or differentiation that are easily confused with genetic structure. To detect such biases, we clustered individuals based on their identity-by-missingness in PLINK v. 1.9 [37,38] where pairwise distances between individuals are calculated from the proportion of missing sites which are not shared between individual pairs. Pairwise distances were visualized with a multidimensional scaling plot.

To estimate and visualize genetic differentiation among individuals, we applied two individual-based clustering methods, STRUCTURE v. 2.3.4 [39] and principal component analysis (PCA) in the R package ade4 [40–42]. STRUCTURE uses model-based Bayesian clustering to find the most probable number of population clusters *K*. Once *K* is defined, it estimates the posterior probability of each individual's genotype to originate in each cluster. STRUCTURE analyses were performed assuming uncorrelated allele frequencies, allowing admixture and with no locprior. The burn-in period was set to 10 000 and the number of Markov chain Monte Carlo (MCMC) repetitions to 50 000. Clusters *K* from 1 to 7 were run three times per *K*. The different runs were merged for visual analysis with CLUMPAK [43]. Calculations of the most probable number of population clusters (*K*) were estimated using STRUCTURE HARVESTER [44] by calculating the posterior probability for each value of *K* (mean lnP(*K*)) and the modal value of Delta *K*. The second individual-based clustering method (PCA) uses a multivariate exploratory approach that makes no prior assumptions about how many populations exist or boundaries between populations. Allele frequencies were centred but not scaled and missing data were replaced by mean allele frequencies with the function scaleGen in ADEGENET [45,46]. PCA was performed using the function dudi.pca in ade4.

#### 2.3.3. Hybridization

To remove potential bias in hybrid analysis, 200 SNPs with the highest overall *F*_ST_ were tested for linkage disequilibrium (LD) in Genepop on the web [47] using 10 000 dememorizations, 100 batches and 5000 iterations per batch. SNPs with significant LD after FDR corrections were removed and replaced with new SNPs until no significant comparisons remained. To assess the accuracy, efficiency and power to correctly identify individuals belonging to different hybrid classes, we used the R package HYBRIDDETECTIVE [48]. We used the function freqbasedsim_AlleleSample to generate three replicates of three simulated data sets with pure parents (Pure_A and Pure_B), first- and second-generation hybrids (F1 and F2) and backcrosses between F1 and pure parents (BC_A and BC_B). The datasets contained 720 individuals and were based on the genotype frequencies from the 200 loci in the western (PureA = Austevoll and Stavanger) and southern (PureB = Kristiansand, Strömstad and Kungsbacka) samples. Simulations were analysed in NEWHYBRIDS v. 1.1 [49] which estimates the posterior probability of each individual to belong to one of the six hybrid classes. The analysis was done using the uniform prior option and default genotype proportions with a burn-in period of 50 000 iteration and 300 000 MCMC sweeps. Power was estimated as the product of efficiency (correctly assigned individuals over the known individuals per class) and accuracy (correctly assigned individuals over individuals assigned to that class) as described in HYBRIDDETECTIVE [48].

Finally, we investigated the occurrence of hybridization in the northern-most location Flatanger in mid-Norway with the software NEWHYBRIDS. Individuals from Skagerrak (Kristiansand, Strömstad and Kungsbacka) and western Norway (Austevoll and Stavanger) were included in the runs as the pure parent genotypes using the ‘z’ and ‘s’ options. The analysis was performed using the same 200 loci as for the simulated data, displaying the highest overall *F*_ST_ estimates and no LD. The data were analysed using the uniform prior option, default genotype proportions and the burn-in period was set to 50 000 and the number of MCMC sweeps after burn-in to 300 000.

## 3. Results

### 3.1. Genetic diversity and population differentiation

From the total 48 technical replicates (four for each pool of 20), we called 237 090 SNPs (average 4939 ± 126 s.d. per replicate pair). Of these, 9% ± 0.05% s.d. were inconsistent between technical replicates. Data filtering resulted in a total of 4372 polymorphic SNPs, and none of the 240 individuals had to be removed due to missing data. Of the 57 600 missing data comparisons, only 479 pairwise comparisons have an identity of missingness higher than 20% (max 47%), and no obvious patterns of identity by missingness can be observed (S3). *F*_IS_ estimates indicate heterozygote excess in all samples (mean *F*_IS_ ranging from −0.344 to −0.052). Fifteen loci deviated significantly (*q* < 0.05) from HW proportions in more than one sample and were subsequently removed, leaving 4357 SNPs for final analysis. No more than eight loci deviate significantly from HW proportions in any of the western or southern samples. However, a much higher number of loci deviate from HW proportions in the Flatanger sample. Almost all of the loci display negative *F*_IS_ values, indicating heterozygosity excess. Furthermore, wrasse from the western population display an overall higher genetic diversity (mean *H*o = 0.30, mean *H*e = 0.32, polymorphic loci = 95.2%) compared with wrasse from the southern population (mean *H*o = 0.26, mean *H*e = 0.24, polymorphic loci = 82.3%). The Flatanger population shows the highest genetic diversity (mean *H*o = 0.50, mean *H*e = 0.35, polymorphic loci = 97%). Global genetic differentiation, estimated as *F*_ST_ = 0.0789, is significantly (*p* < 0.05) different from zero. Pairwise *F*_ST_ estimates (S4) demonstrate higher genetic differentiation between the western and southern populations (*F*_ST_ = 0.101–0.1312) than among the southern samples (*F*_ST_ = 0.0023–0.0030) or between the western samples (*F*_ST_ = 0.0065). Overall, Flatanger is genetically more similar to the western population (*F*_ST_ = 0.0243–0.0277) than the southern population (*F*_ST_ = 0.1163–0.1258).

### 3.2. Individual-based clustering

**Figure 2.**
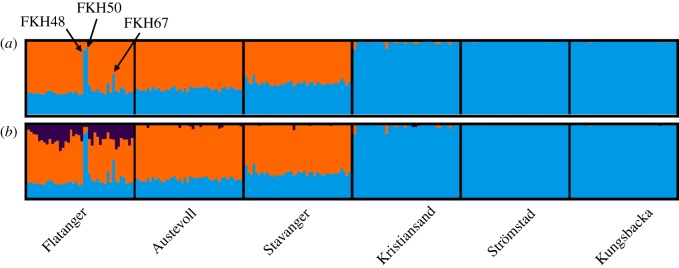
STRUCTURE cluster assignment of corkwing wrasse based on 4357 SNPs for *K* = 2 (*a*) and *K* = 3 (*b*). Each vertical line represents one individual and the colour shows the proportion of each individual assigned to the *K* different genetic clusters. Individuals from Skagerrak/Kattegat cluster together (blue) and individuals from western Norway cluster together (orange), visualizing the genetic break between southern and western populations. Majority of individuals in mid-Norway (Flatanger) cluster with the western population, with the exception of FKH48 and FKH50, which cluster with southern population, and FKH67, which does not group to either cluster.

STRUCTURE analyses suggest the existence of two, potentially three, genetically differentiated clusters (figure 2; electronic supplementary material, figure S5). The first two clusters correspond to the divide between southern and western populations (blue and orange, respectively), in concordance with pairwise *F*_ST_ estimates and previous studies [19]. Most individuals from Flatanger were assigned to the western population for *K* = 2, and partially to a third cluster (purple) for *K* = 3. However, two individuals from Flatanger (FKH48 and FKH50) were assigned to the southern population (blue). Another individual from the Flatanger sample (FKH67) was assigned equally to both populations, suggesting admixture.

**Figure 3.**
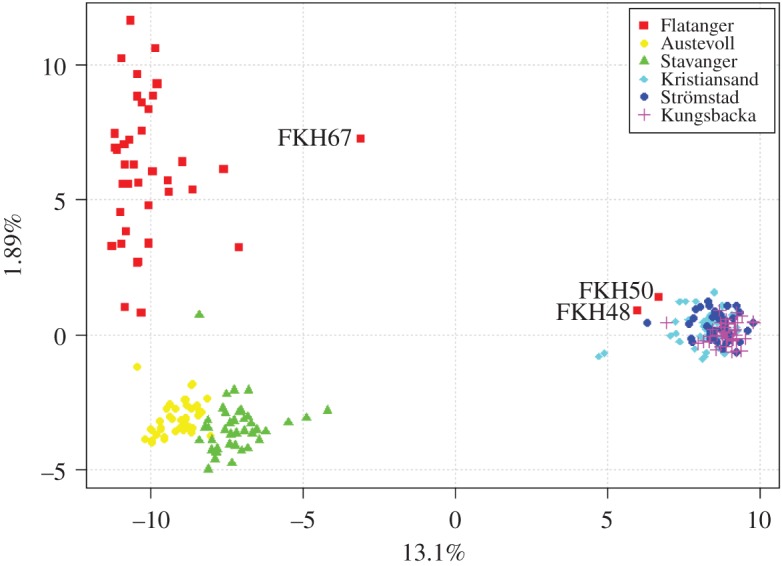
The first (*x*-axis) and second (*y*-axis) components of a principal component analysis on 240 corkwing wrasse individuals from 6 locations based on 4357 SNPs. The first component explains 13.1% of the total variation and the second 1.89%. Additional components explain less than 1% of the total variance each, and are not shown. Each point represents one individual, which is colour coded by sampling site. On the first axis, majority of individuals from Flatanger cluster with individuals from western Norway (left), but two individuals from Flatanger (FKH48 and FKH50) cluster together individuals from Skagerrak/Kattegat (right) and one individual (FKH67) separates from both clusters. On the second axis, individuals from Flatanger are more separated, but overall closer to Skagerrak/Kattegat than western Norway.

To estimate and visualize genetic differentiation among individuals without prior assumptions about the population model, we conducted a PCA (figure 3). The first principal component separates data into two main clusters, which correspond closely to southern and western clusters observed in the STRUCTURE analysis. The second principal component (*y*-axis) splits the Flatanger population from the western population, placing Flatanger closer to the southern population than the western. Succeeding components explain less than 1% of the total variance each, and are not shown. The same two individuals from Flatanger (FKH48 and FKH50) which were assigned to the southern cluster in the STRUCTURE analyses group with the southern cluster in the PCA. The Flatanger individual which was assigned equally to both clusters in the STRUCTURE analyses (FKH67) is closer to the southern cluster than any of the other individuals from the Flatanger or western population.

### 3.3. Hybridization

**Figure 4.**
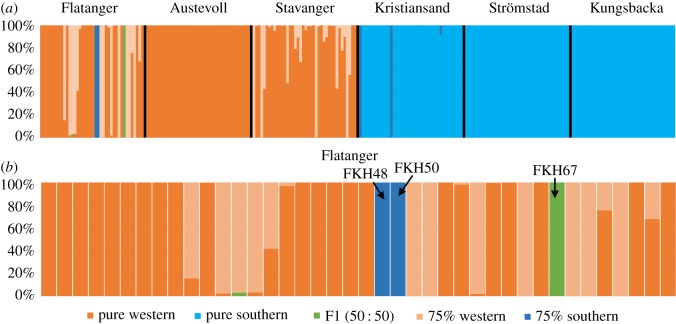
Hybrid analysis of all individuals (*a*) and individuals sampled in Flatanger (*b*) using the 200 SNPs with highest *F*_ST_ estimates and no LD. Each vertical line represents one individual and its probability belongs to one of the six genotype classes: pure western, pure southern, F1 hybrid (50 : 50 western:southern), F2 hybrid (none present) or backcrosses (75% western or southern) between F1 and pure western or pure southern. Hybrids are only detected in Flatanger and the two samples next to the genetic break, Stavanger and Kristiansand. Out of the 40 individuals from Flatanger, we discovered two individuals with clear southern genotypes (FKH48 and FKH50), one first-generation hybrid (FKH67), and twelve potential second-generation hybrids (light orange).

**Figure 5.**
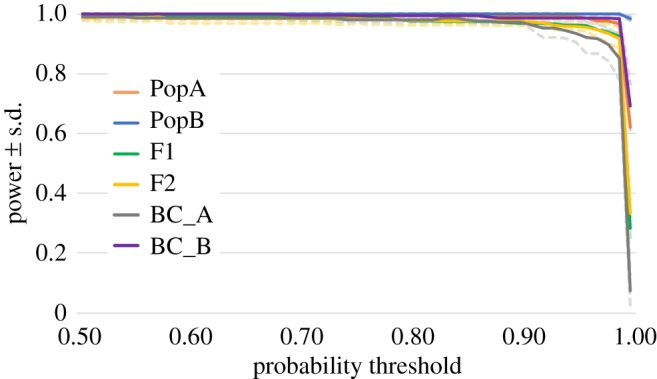
Hybrid detection power at different probability thresholds based on three sets of simulated genotype data from 200 SNPs with the highest overall *F*_ST_ and no LD. Solid lines represent the six genotype classes, pure parents (PopA = western population and PopB = southern population), first- and second-generation hybrids (F1 and F2) and backcrosses (BC_A and BC_B). The dashed lines represent the standard deviation among the simulations for each class.

We used the software NEWHYBRIDS to identify potential hybrids in Flatanger (figure 4). The two individuals, assigned to the southern cluster in both STRUCTURE and the PCA (FKH48 and FKH50), were identified as southern backcrosses, i.e. 75% southern genotype and 25% western genotype. FKH67 was detected as a F1 hybrid, carrying 50% of both the southern and western genotype. Furthermore, another 12 individuals from Flatanger have a high probability (greater than 50%) of being western backcrosses, i.e. 75% western genotype and 25% southern genotype. Some fish from the ‘pure’ southern and western samples closest to the southern/western genetic break (two individuals in Kristiansand and eight in Stavanger) are also distinguished as genetic backcrosses (figure 4*a*), indicating gene flow across the break. Simulated data demonstrated high efficiency, accuracy and power to detect individuals from all of the six hybrid classes given the battery of 200 loci used (figure 5; electronic supplementary material, figure S6). The battery of SNPs is able to call individuals as pure western, pure southern, F1, F2, western or southern backcross with a power above 95% at a probability threshold of 90%.

**Figure 6.**
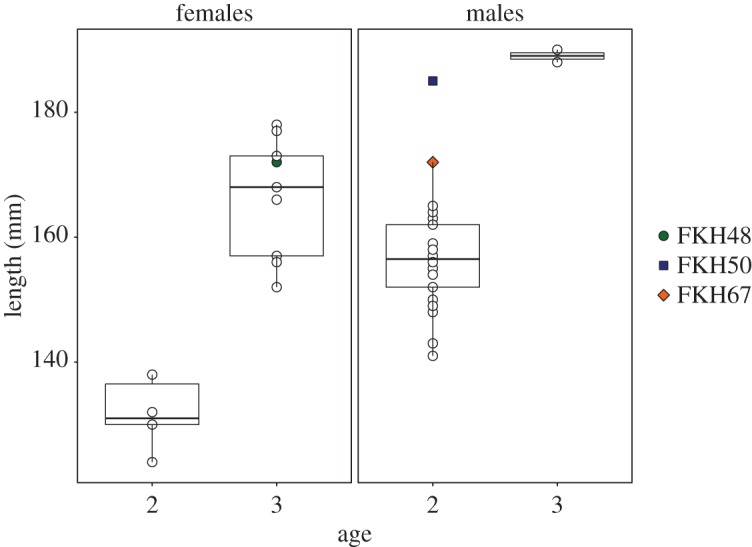
Boxplots showing length at age for corkwing wrasse sampled in Flatanger. FKH48, FKH50 and FKH67 are individuals with genotypes closely resembling southern populations.

Comparison of length measurements for individuals of the same sex and age (figure 6) shows that the F1 individual (FKH67) and one of the individuals with southern genotype (FKH48) are the largest 2-year-old males in the sample. The second individual with a southern genotype (FKH50) is a 3-year-old female above median length.

## 4. Discussion

Here, we provide the first evidence that translocated corkwing wrasse escape salmon farms and hybridize with local populations. Our results support previous studies by finding marked genetic differentiation between southern Skagerrak corkwing wrasse populations and those in western Norway. We expand on current knowledge by discovering that almost half of the individuals sampled at the northern limit of the species distribution range have partial southern genotypes. Three of these individuals carry 50% or more of the southern genotype. We discuss the potential consequences of human-mediated gene flow and the concerns with the current practice of large-scale translocation of wrasse.

### 4.1. Population diversity and differentiation

As expected under isolation by distance, pairwise *F*_ST_ estimates (S2) demonstrate that the Flatanger as a whole is genetically most similar to Austevoll, followed by Stavanger, while almost 10-fold more differentiated from the southern sampling locations. We observe similar patterns of genetic differentiation in the individual-based clustering methods for a majority of individuals from Flatanger (figures 2 and 3). This suggests that the Flatanger population is largely a result of an ongoing northward range expansion, as suggested by Knutsen *et al.* [24]. It is possible that a more continuous sampling along the west coast of Norway would have improved upon these results by adding samples closer to Flatanger, and hence more likely to have contributed to a range expansion.

While we find a clear western/southern genetic break and an overall lower genetic diversity in the southern, Skagerrak region [19,24], the highest diversity can be seen in Flatanger, which is rather surprising, considering that this area has been colonized recently [19,23,24] and is on the leading edge of a range expansion [19,24]. Typically, a reduction in genetic diversity is to be expected when a species colonizes a new area [50]. The high genetic diversity in Flatanger is, therefore, likely to be a result of multiple sources of origin and recent interbreeding [50], as indicated by the fact that roughly 40% of all loci demonstrated a significant heterozygosity excess in the Flatanger sample.

### 4.2. Hybridization

Two individuals (FKH48 and FKH50) exhibit high similarity to the southern population while differentiating from all western and Flatanger individuals. They clearly cluster with southern individuals in STRUCTURE and PCA, suggesting a southern genotype. A third individual (FKH67) did not cluster with either southern or western populations, and was classified as a F1 hybrid (50 : 50 western:southern) by NEWHYBRIDS (figure 4). Furthermore, NEWHYBRIDS found twelve Flatanger individuals to have more than 50% probability of being western backcrosses. This strongly supports ongoing hybridization between the southern and western genotypes in the wild, which has previously only been documented in captivity [26]. We also detected two potential backcrosses in Kristiansand and seven in Stavanger (figure 4*b*) in addition to the hybrids discovered in Flatanger. Stavanger and Kristiansand are the two samples collected closest to the genetic break on the western and the southern side, respectively. Except for Flatanger, we did not detect any indication of hybrids in any of the other samples further from the genetic break, indicating the existence of isolated populations [19].

The relatively high number of southern--western hybrids in Flatanger is, therefore, convincing evidence of escapement and hybridization of cleaner fish sourced from Skagerrak and/or Kattegat. Recently, Jansson *et al*. [18] showed there to be much lower differentiation than expected in goldsinny wrasse between Flatanger and Skagerrak populations indicating escapees and possibly hybridization. Unfortunately, there are no official records on the locations of source and destination of wrasses used as cleaner fish, which could have facilitated further interpretation of these results. Upon consulting with the four wrasse transport companies, they confirmed that the clear majority of wrasse being translocated in Norway are exported from Skagerrak–Kattegat coast to farms in mid- and northern Norway. Furthermore, translocations of wrasse from western Norway to mid-Norway have been strongly discouraged by food-safety authorities due to the possibility of wrasse being a carrier of pancreas disease which affects farmed salmon and is endemic in western Norway south of Hustadvika [51]. Combined, this supports the conclusion that western backcrosses in Flatanger must have been the result of hybridization with southern genotypes from Skagerrak and/or Kattegat. We did not find any western backcrosses east of Kristiansand in the Skagerrak. Consequently, the western backcross genotypes we found in Flatanger are likely a result of second-generation hybridization that occurred after translocation. Two of the companies reported to also have transported wrasse from Skagerrak to farms in western Norway. Thus, it is presently unclear whether the occurrence of western backcrosses in the Stavanger area is a result of human-mediated translocation, or if it is due to occasional natural gene-flow across the genetic break between the southern and western populations.

The onset of gene flow between previously isolated populations may have genetic, physiological and ecological consequences. The corkwing wrasse in Flatanger most likely colonized the area within the last two decades [23]. This and low catch rates attest to a very low abundance in the Flatanger area compared to regions further south (Per Andersen 2016, personal observation), rendering this population more vulnerable to hybridization events. Presently, fishing for wrasse in Sweden is allowed from 15 May, and occurs during their spawning period in May and June [52]. Hence, there is a possibility that ready-to-spawn corkwing are escaping during the spawning season, increasing the probability of hybridization. In Norway, the wrasse fishery is closed until the end of the spawning season [22], which reduces the chances of hybridization. In the UK, wrasse fishery has no temporal restrictions nationally, but in 2017 three southwestern IFCAs implemented byelaws that restrict wrasse fishery to certain periods of the year in specified areas [15–17].

### 4.3. Implications

The effects of hybridization between genetically distinct populations are hard to predict and depend on many factors. Fitness can increase as a result of introducing favourable alleles and genotypes (overdominance), or because of deleterious alleles being sheltered (heterosis) [50]. The three individuals with more than 50% southern genotype tended to be larger than the native fish at the same age. Although a conclusion cannot be reached without a larger sample size of hybrids, this is consistent with earlier findings of southern corkwing growing faster than western [22]. If the faster growth and larger body size for southern populations have a genetic basis, hybrids may have a fitness advantage in reproduction, either through sexual selection for large males or higher fecundity of large females. Alternatively, a reduction in fitness can occur due to genetic incompatibilities (intrinsic outbreeding depression) or reduced adaptation to the local environment (extrinsic outbreeding depression) [50]. The life history differences between southern and western populations have been suggested to reflect temperature differences between these regions [22]. If there is local adaptation, it is likely that the continued transfer of unfit individuals would cause the loss of locally adapted alleles and genotypes, known as genetic swamping [53]. However, introgression and admixture of the southern genotype into the Flatanger population are likely to continue, whether there is an increase of fitness or not. This is because all of the hybrids' progeny will also be hybrids [50].

Populations on the boundary of a species range exist in conditions similar to the habitats just outside the distribution range, making them more likely to carry genotypes that are able to colonize new habitats [54]. As the Flatanger population constitutes the northern boundary of the species distribution, it is likely to play an important role for future adaptation potential, and range expansion. However, the asymmetric gene flow to the edges of a species range can obstruct this adaptation [55]. Admixing with southern genotypes might, therefore, work as a barrier to further range expansion. Furthermore, southern corkwing is also translocated to salmon farms even further north, to the Nordland county (Jacob Meland, Lovundlaks 2017, personal communication), where no wild corkwing populations are present. This could facilitate further spreading of southern genotypes beyond the current natural range. In addition to the genetic and ecological risks discussed above, escaping wrasse may introduce new diseases or parasites to conspecifics, salmon and other species in the wild [13,56,57]. Murray [58] argues that the risks of disease transfer from cleaner fish to salmon are small compared to the risk posed by sea lice, but disease transfer to the local populations of wrasse and other species was not considered. With ongoing hybridization, the risk of disease transfer may be an even greater threat to local populations, because hybrids may be more susceptible to diseases and parasites, as seen in other fish species [59,60].

In the face of climate-induced changing environments, conservation of populations on the leading edge should be prioritized to maximize future adaptive potential [54,61,62]. We argue that any evaluation of the risks with the translocation of wrasse needs to include effects on wild populations and ecosystems. However, prohibiting long-distance transport and sourcing wrasse locally might also pose a problem as local stocks are prone to overexploitation [12,22,63]. An obstacle for effective management is that the current practice of cleaner fish use is poorly documented and regulated. Norwegian law states that aquacultures are obligated to report all escaping fish from aquaculture installations, but presently only the target species cultured are recorded. Moreover, Norwegian and UK transporters are not required to log and report the source or the destination of cleaner fish, which complicates the possibilities to assess and address the problem of escapees.

## 5. Conclusion

We provide the first evidence that translocated wild corkwing wrasse used as cleaner fish in salmon farms escape and hybridize with local populations at the northern limit of its distribution. These findings provide important information for aquaculture management and conservation of wild populations of non-target species, and have implications for the increasing use of cleaner fish as parasite control in fish farms, which is both poorly documented and regulated. Moving genetic material between isolated populations could drastically alter the genetic composition, erode population structure and potentially result in loss of local adaptation, hampering the species expansion. The geographical extent and magnitude of introgression and the ecological consequences remain unknown for this and other wrasse species. It is urgent to address these gaps of knowledge, as there is no immediate sign of reduction of the current practice in Norway, and wrasse are increasingly being deployed in other areas such as the UK.

## Supplementary Material

Electronic Supplementary Material S1-S6 from Cleaner fish escape salmon farms and hybridize with local wrasse populations

## Data accessibility

Data are deposited on Dryad: http://dx.doi.org/10.5061/dryad.tv553 [64]. Raw data are available on NCBIs Sequence Read Archive (BioProject PRJNA415388). Scripts used for bioinformatic analysis can be found at https://github.com/z0on/2bRAD_denovo. Log of the pipeline used can be found at https://github.com/ellikafaust/S.melopsPopGen/blob/master/2bRAD_log_escapee.
