# Supplementary material for: Cleaner fish escape salmon farms and hybridize with local wrasse populations
Source: R Soc Open Sci. 2018 Mar 21;5(3):171752. doi: 10.1098/rsos.171752 (PMC5882703; doi:10.1098/rsos.171752)
Supplement: Electronic Supplementary Material S1-S6 from Cleaner fish escape salmon farms and hybridize with local wrasse populations [file rsos171752supp1.doc]

# Supplementary

S1 Sampling information of corkwing wrasse (*Symphodus melops)*. Sample size indicates the number of individuals collected at each site.

| Sample name | Area | Sample size | Year | Area Coordinates |
| --- | --- | --- | --- | --- |
| Flatanger | Norwegian Sea | 40 | 2016 | 64.513943, 10.679272 |
| Austevoll | North Sea N | 40 | 2016 | 60.096642, 5.269618 |
| Stavanger | North Sea S | 40 | 2016 | 58.963804, 5.942488 |
| Kristiansand | Skagerrak W | 40 | 2016 | 58.187534, 8.048447 |
| Strömstad | Skagerrak E | 40 | 2016 | 58.947222, 11.000000 |
| Kungsbacka | Kattegat | 40 | 2016 | 57.403142, 11.907473 |

S2 Map of sampling locations in Flatanger and the nearby open pen Salmon farms (data from https://kart.fiskeridir.no/akva). Left: Sampling locations of Western backcrosses (black) and Southern backcrosses (coloured). Right: Sampling locations of Western genotypes (open circles). The size of the circles corresponds to the number of individuals at each of the locations 1-5.) Visualisation done on http://www.copypastemap.com.

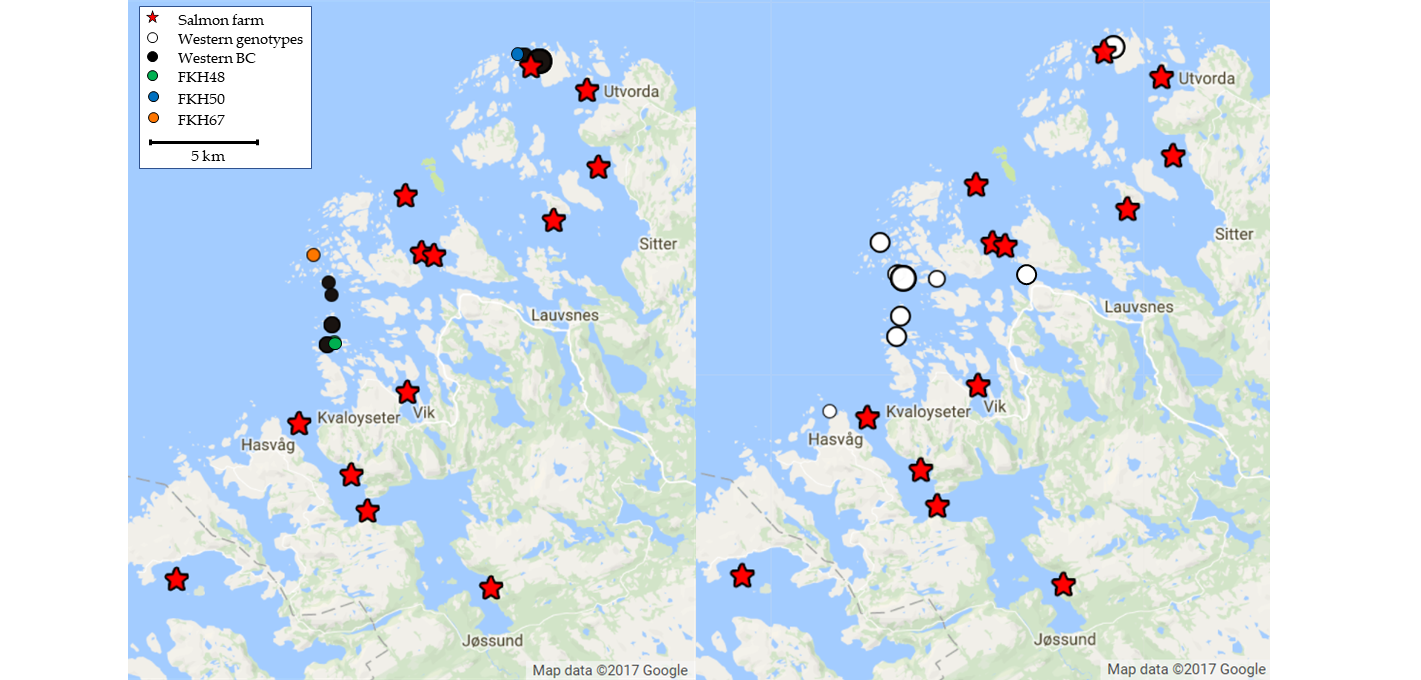


S3 Multidimensional scaling plot showing identity by missingness calculated in PLINK [1]. First (x-axis) and second (y-axis) dimensions of 240 corkwing wrasse individuals from 6 locations based on identity by missingeness in 4357 SNPs. Each point represents one individuals, which are colour coded by sampling site. No clear structure or pattern of missingness can be seen.

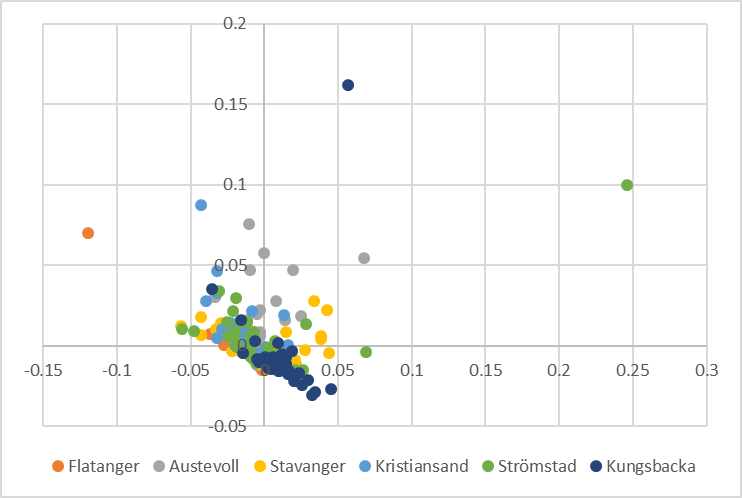


S4 Matrix of pairwise F_ST_ according to Weir and Cockerham (1984) calculated with the R package diveRsity [2]. Significance of the estimates was tested using Fisher's Exact tests with 10000 MC reps. * q < 0.001

|  | Flatanger | | Austevoll | Stavanger | Kristiansand | Strömstad | Kungsbacka |
| --- | --- | --- | --- | --- | --- | --- | --- |
| Flatanger | |  |  |  |  |  |  |
| Austevoll | | 0.0243* |  |  |  |  |  |
| Stavanger | | 0.0277* | 0.0065* |  |  |  |  |
| Kristiansand | | 0.1163* | 0.1213* | 0.101* |  |  |  |
| Strömstad | | 0.1218* | 0.1281* | 0.1072* | 0.0023 |  |  |
| Kungsbacka | | 0.1258* | 0.1312* | 0.1102* | 0.0030 | 0.0029 |  |

S5 A visualisation of mean LnP(K) ± SD and delta K for K 1-7 clusters run 3 times in STRUCTURE [3]. Values calculated using Structure Harvester, http://taylor0.biology.ucla.edu/structureHarvester [4].


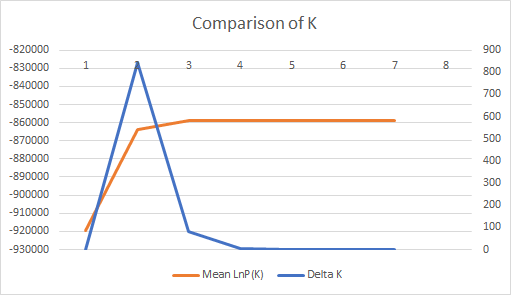


S6 Assignment efficiency and accuracy at different probability thresholds based on three sets of simulated genotype data [5] from 200 SNPs with the highest over all *F*_ST_ and no LD. Solid lines represent the 6 genotype classes, pure parents (PopA = western population and PopB = southern population), first and second-generation hybrids (F1 and F2) and backcrosses (BC_A and BC_B). The dashed lines stand for the standard deviation among the simulations for each class. Efficiency = correctly assigned individuals over the known individuals per class. Accuracy = correctly assigned individuals over individuals assigned to that class.


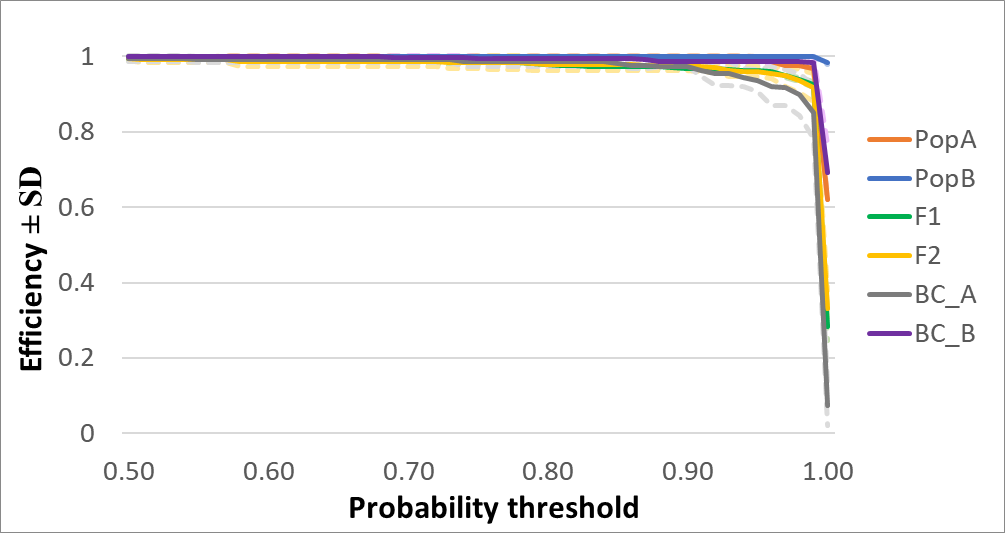


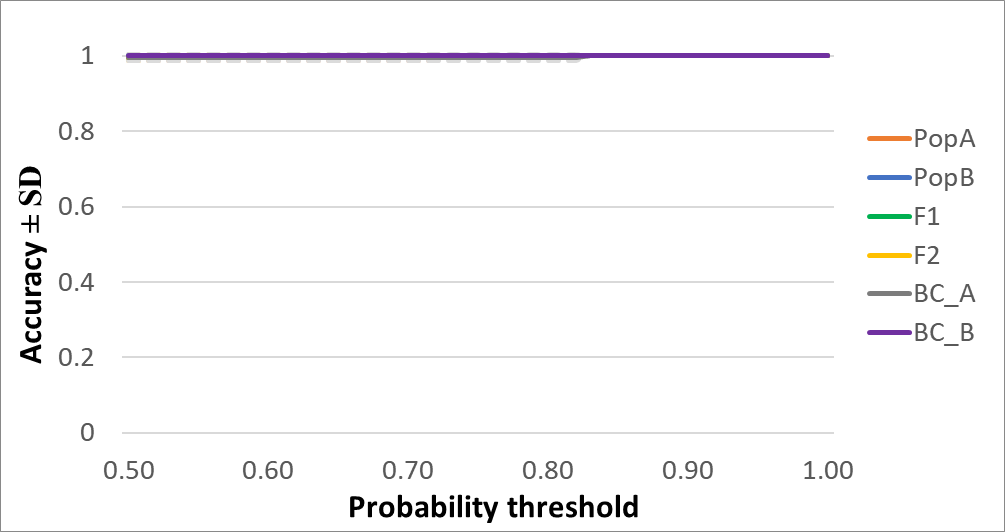


1. Chang CC, Chow CC, Tellier LCAM, Vattikuti S, Purcell SM, Lee JJ. 2015 Second-generation PLINK: Rising to the challenge of larger and richer datasets. *Gigascience* **4**, 1–16. (doi:10.1186/s13742-015-0047-8)

2. Keenan K, Mcginnity P, Cross TF, Crozier WW, Prodöhl PA. 2013 diveRsity: An R package for the estimation and exploration of population genetics parameters and their associated errors. *Methods Ecol. Evol.* **4**, 782–788. (doi:10.1111/2041-210X.12067)

3. Pritchard JK, Stephens M, Donnelly P. 2000 Inference of population structure using multilocus genotype data. *Genetics* **155**, 945–959. (doi:10.1111/j.1471-8286.2007.01758.x)

4. Earl DA, Von Holdt BM. 2012 STRUCTURE HARVESTER: a website and program for visualizing STRUCTURE output and implementing the Evanno method. *Conserv. Genet. Resour.* **4**, 359–361. (doi:10.1007/s12686-011-9548-7)

5. Wringe BF, Stanley RRE, Jeffery NW, Anderson EC, Bradbury IR. 2017 HYBRIDDETECTIVE: A workflow and package to facilitate the detection of hybridization using genomic data in R. *Mol. Ecol. Resour.* **17**, e275–e284. (doi:10.1111/1755-0998.12704)
